# Fabrication, Characterization, and In Vitro Cytotoxicity Assessment of Tri-Layered Multifunctional Scaffold for Effective Chronic Wound Healing

**DOI:** 10.3390/bioengineering10101148

**Published:** 2023-09-30

**Authors:** Ahmed Olanrewaju Ijaola, Balakrishnan Subeshan, Anh Pham, Md. Nizam Uddin, Shang-You Yang, Eylem Asmatulu

**Affiliations:** 1 Department of Mechanical Engineering, Wichita State University, 1845 Fairmount St., Wichita, KS 67260, USA; aoijaola@shockers.wichita.edu (A.O.I.); bxsubeshan@shockers.wichita.edu (B.S.); akpham@shockers.wichita.edu (A.P.); 2 Department of Biological Sciences, Wichita State University, 1845 Fairmount St., Wichita, KS 67260, USA; 3 Department of Engineering and Physics, Texas A&M University-Texarkana, 7101 University Ave, Texarkana, TX 75503, USA; muddin@tamut.edu; 4 Department of Orthopedic Surgery, University of Kansas School of Medicine-Wichita, Wichita, KS 67214, USA

**Keywords:** chronic wounds, nanobiotechnology, biomaterials, nanofibrous scaffolds, tri-layered scaffolds, electrospinning, hydrogel

## Abstract

Chronic wounds have been a global health risk that demands intensive exploration. A tri-layered biomaterial scaffold has been developed for skin wounds. The top layer of the scaffold is superhydrophobic, and the bottom layer is hydrophilic, both of which were electrospun using recycled expanded polystyrene (EPS) and monofilament fishing line (MFL), respectively. The intermediate layer of the scaffold comprised hydrogel by cross-linking chitosan (CS) with polyethylene glycol. The surface morphology, surface chemistry, thermal degradation, and wettability characteristics of each layer of the scaffold were examined. Also, the antibacterial activity and in vitro cytotoxicity study on the combined tri-layered scaffold were assessed against *Escherichia coli* (*E. coli*) and *Staphylococcus aureus* (*S. aureus*). Data revealed exceptional water repellency of the heat-treated electrospun top superhydrophobic layer (TSL) with a high-water contact angle (WCA) of 172.44°. A TSL with 15 wt% of micro-/nano-inclusions had the best thermal stability above 400 °C. The bottom hydrophilic layer (BHL) displayed a WCA of 9.91°. Therapeutically, the synergistic effect of the combined tri-layered scaffold significantly inhibited bacteria growth by 70.5% for *E. coli* and 68.6% for *S. aureus*. Furthermore, cell viability is enhanced when PEG is included as part of the intermediate CS hydrogel layer (ICHL) composition.

## 1. Introduction

Human skin serves as a barrier protecting internal organs from external damage and is an organ of sensory, fluid homeostasis, and a selectively permeable layer to excretion/absorption. However, being exposed to the environment causes human skin to be vulnerable to injury. Cuts and minor injuries on the human skin can be healed due to its regenerative characteristics, but this process can be impaired, thereby prolonging the healing process [1]. A significant and recurrent complication arises in the form of bacterial infections at the wound site, which impede the overall pace of healing. Due to the presence of bacteria that adhere to the wound site, these infections can grow and multiply as a unicellular organism, thereby giving rise to acute infections. Alternatively, they may colonize the skin’s surface as an agglomerate, which poses the risk of transforming into a chronic wound [2,3]. This occurrence affects approximately 6.5 million people in the United States, and the numbers are expected to increase due to the increases in obesity, diabetes, prediabetes, injured veterans, an aging population, and lack of healthcare in rural areas. In the United States, treatment for chronic wounds costs around $25 billion [4,5]. There is a call for advanced techniques and materials to treat chronic wounds effectively at a low cost to prevent this silent epidemic.

Various treatment approaches for chronic wound healing have been employed, including innovative nano-therapy, ozone treatment, ulcer care, acute care surgery, and high-dose antibiotics. In recent years, biomaterials have emerged as a promising avenue for accelerating the healing process of chronic wounds [6,7]. Among various biomaterial categories, ideal scaffolds should mimic the components of the natural extracellular matrix (ECM) of the skin that exhibits a highly interconnected porous structure to facilitate enhanced cell adhesion integration, and offer added benefits of controlled degradation and incorporating bioactive agents of promoting chronic wound healing [8]. Scaffolds for chronic wound healing and skin regeneration can be fabricated into single-layer, bi-layer, or tri-layer scaffolds [9]. According to Haldar et al., there has not been a successful single-layer or bi-layer scaffold engineered for skin regeneration due to a lack of water content in the scaffold, the abrupt hydrophobicity–hydrophilicity transition between the layers [10].

The tri-layered scaffold design and fabrication represent crucial aspects of biomaterial research and may play a pivotal role by providing essential support for cellular proliferation while preserving their specialized functions, indicating some idea characteristics for skin wound healing. The architecture of the tri-layered scaffold serves as a defining factor in achieving the desired final shape, which comprises three distinct layers. The top layer of the scaffold should be designed to have minimal porosity, maximizing its hydrophobic nature while resembling the characteristics of the epidermis. The intermediate layer should be constructed to possess increased porosity and a slightly hydrophilic nature, promoting the regeneration of the dermal layer. The purpose of the bottom layer is to facilitate subcutaneous regeneration by effectively retaining a copious amount of moisture. Therefore, the fabrication process of the bottom layer should be optimized to ensure that it exhibits the highest hydrophilicity among the three layers [11,12].

When developing a tri-layered scaffold for chronic wound healing, it is crucial to ensure that it possesses a range of essential functionalities. These functionalities include the ability to facilitate fluid exchange, maintain a moist environment to prevent excessive heat buildup at the wound site, provide a protective barrier against bacterial infections, exhibit non-toxic properties, absorb wound odors, promote biocompatibility, minimize the risk of allergic reactions, prevent scarring, and maintain sterility [13,14,15,16,17]. However, available tri-layered scaffolds often suffer from deficiencies in mechanical strength, insufficient protection of the wound surface against microbial infection and external influences, limited adjustability in terms of adhesion strength, and lack of precise control over drug release at the wound site [18,19,20].

The current study reports on the fabrication of a novel multifunctional and biomimetic tri-layered scaffold with bioactive chitosan (CS) hydrogel sandwiched between electrospun top superhydrophobic and bottom hydrophilic nanofibrous layers from waste polymers for chronic wound healing. The rationale of the fabrication was to mimic specific layers of the skin. The epidermal layer of the skin acts as the outermost protective barrier for the internal organs. The epidermis is characterized by its high hydrophobicity, which prevents moisture loss from the underlying layers [21]. The top layer of the scaffold was prepared using EPS/PTFE-µPs/SiO_2_-NPs to emulate this hydrophobic nature to enable effective moisture retention within the scaffold. The beneath dermis layer of the skin exhibits a relatively higher level of porosity to facilitate cell growth and tissue regeneration [22]. A chemically cross-linked CS intermediate hydrogel layer is formed to mimic the dermis since CS can provide the necessary strength while providing an interconnected porous structure. The bottom layer of the scaffold plays a crucial role in emulating the characteristics of the bottom hypodermal layer of the skin as well as serving as a self-adhesive base, facilitating the adherence of the scaffold to the wound bed [23]. Moreover, this layer is engineered to exert an inhibitory effect on bacterial growth upon contact, thus minimizing the risk of infections [24]. MFL/HAp/Ag-NPs were selected for synthesizing the bottom layer of the scaffold. The objectives of this investigation were to characterize the combination of electrospun nanofibrous layers and bioactive CS hydrogel in the engineered tri-layered scaffold and evaluate its nature of the microbial inhibition and tissue biocompatibility.

## 2. Materials and Methods

### 2.1. Materials

Expanded polystyrene waste and monofilament fishing line were recycled and utilized without additional purification. Polytetrafluoroethylene microparticles (PTFE-µPs) (particle size = 3 µm, M_w_ = 678.10 gmol^−1^), silicon dioxide nanoparticles (SiO_2_-NPs) (particle size = 60–70 nm, M_w_ = 100 gmol^−1^), and silver nanoparticles (Ag-NPs) (99.99%, 20 nm, metal basis) were purchased from U.S. Research Nanomaterials Inc. (Houston, TX, USA). Hydroxyapatite (HAp) (<200 nm, M_w_ = 502.31 gmol^−1^) was purchased from Sigma-Aldrich (St. Louis, MO, USA). The chemical reagents N, N-Dimethylformamide (DMF) (C_3_H_7_NO, 99.8%), acetone (C_3_H_6_O), and acetic acid (CH_3_COOH) were purchased from Thermo Fisher Scientific (Waltham, MA, USA). Formic acid (CH_2_O_2_, 99%) was purchased from Manofohm Chemicals (Florence, CO, USA). Chitosan powder was procured from Chemsavers (90+% deacetylated) (Bluefield, VA, USA). Polyethylene glycol (M_w_ = 7000–9000 gmol^−1^) was purchased from HiMedia Laboratories (Kennett Square, PA, USA). Glycerol phosphate disodium salt (isomeric mixture of 50% β-isomer and 50% rac-α-isomer) was obtained from GTI Laboratory Supplies (Edna, TX, USA). Bovine serum albumin (98% purity) was purchased from Protein Mods, LLC (Waunakee, WI, USA). Aloe vera leaf juice (99.5% purity) was purchased from Nature’s Way Brands, LLC (Green Bay, WI, USA). *E. coli* and *S. aureus* were obtained from Sigma-Aldrich (St. Louis, MO, USA). Fibroblast cell lines (3T3 and L-929 cells) were obtained from American Type Culture Collection (ATCC) (Manassas, VA, USA) and cell culture medium and reagents were from ThermoFisher Scientific (thermofisher.com, accessed on 30 March 2021) and ScienCell Research Laboratories (Carlsbad, CA, USA). The chemical reagents and cell medium were employed in their original form without any modifications or additional purification steps.

### 2.2. Fabrication of Tri-Layered Scaffold

To fabricate the top superhydrophobic layer (TSL) of the tri-layered scaffold, small pieces of EPS waste were dissolved in the DMF and C_3_H_6_O solvent mixture at a solvent-to-polymer ratio of 80:20. Then, the total amount of PTFE-µPs and SiO_2_-NPs were added at 5, 10, and 15 wt% to the EPS/DMF/C_3_H_6_O mixture. The resulting EPS/PTFE-µPs/SiO_2_-NPs polymeric solution was stirred continuously for 6 h at 50 °C. Next, sonication was carried out for 15 min using a probe sonicator to ensure a homogeneous solution. The bottom hydrophilic layer (BHL) of the tri-layered scaffold was electrospun with MFL waste. First, small pieces of MFL waste were dissolved in a solvent mixture of CH_2_O_2_ and CH_3_COOH (ratio of 4:1) at a solvent-to-polymer ratio of 83:17. HAp and various weight percentages of Ag-NPs (0.1, 0.4, 0.8, and 1.5 wt%) were then applied to the MFL/CH_2_O_2_/CH_3_COOH mixture. The resulting MFL/HAp/Ag-NPs solution was stirred for 36 h at ambient temperature to obtain a homogeneous solution.

The prepared EPS/PTFE-µPs/SiO_2_-NPs solution (for TSL) and MFL/HAp/Ag solution (for BHL) were loaded separately into 10 mL syringes with a blunt metal needle of 0.5 mm diameter and electrospun at a constant rate of 2 mL/h. The spinning distance, applied voltage, temperature, and relative humidity were controlled at 25 cm, 25 kV, 21 ± 5 °C, and 46 ± 5%, respectively. These process parameters were kept consistent for all electrospun nanofibrous layers. All electrospun nanofibrous layers were left on the aluminum screen for 12 h to dry. Finally, the electrospun EPS/PTFE-µPs/SiO_2_-NPs nanofibrous TSLs were further dried in an electric oven at different temperatures (50 °C, 70 °C, 90 °C, and 110 °C) for 6 h. The electrospun MFL/HAp/Ag-NPs nanofibrous BHLs were vacuum dried at 38 °C for five days to remove all trapped moisture and residual solvent.

**Figure 1:**
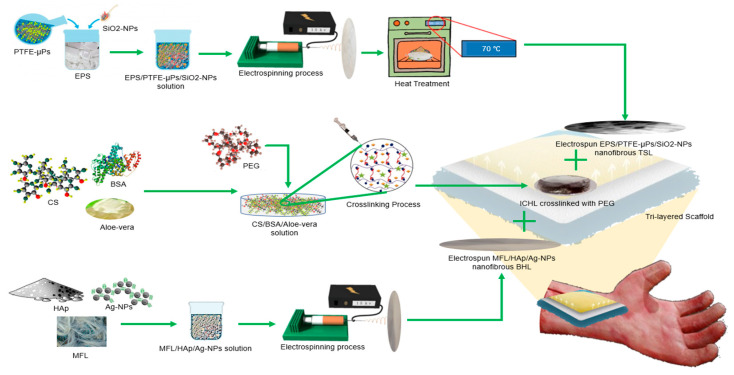
Schematic diagram demonstrating preparation of tri-layered scaffold: TSL resembles epidermis, ICHL promotes regeneration of dermal layer, and BHL emulates dermal structure of human skin.

The intermediate CS hydrogel layer (ICHL) was made by dissolving 3.0 g of CS in 0.05 M CH_3_COOH (100 mL) with stirring for 12 h at ambient temperature. The resulting CS/CH_3_COOH solution was sonicated for 20 min to remove possible trapped air bubbles. Next, a pre-mixed BSA (1.5% w/v), glycerol phosphate disodium salt (1% w/v), and aloe vera (1% v/v) were stirred into CS/CH_3_COOH solution at 60 °C to produce an ICHL sample without PEG. Additionally, PEG (0.5% w/v) was added to the CS solution to produce an ICHL sample with PEG. Finally, the ICHL samples were cured in an oven at 37 °C for 12 h. A schematic diagram of the fabrication process for the tri-layered scaffold is shown in Figure 1.

### 2.3. Characterization of Tri-Layered Scaffold

The surface morphologies of all three fabricated layers of the tri-layered scaffold were characterized using an FEI Nova Nano 450 scanning electron microscope (SEM) provided by the Nebraska Center for Materials and Nanoscience, Nebraska-Lincoln, USA. Fourier transform infrared (FTIR) spectra for all three fabricated layers were measured using a Nicolet^TM^ iS50 (Thermo Fisher Scientific, Lenexa, KS, USA) infrared microscope, performing 128 scans on average and a resolution of 4 cm^−1^ and covering a spectral range of 15–27,000 cm^−1^ using the attenuated total reflectance mode. Thermogravimetric analysis (TGA) for all three fabricated layers was conducted under a nitrogen gas atmosphere on a Q5000 thermogravimetric analyzer (Nebraska Center for Materials and Nanoscience, Nebraska-Lincoln, USA) at a heating rate of 10 °C/min and a flow rate of 60 mL/min. Water contact angles of TSL and BHL of the tri-layered scaffold were measured at six different locations of each surface layer using a contact angle goniometer (KSV Instruments Ltd., Helsinki, Finland, Model #CAM 100) to obtain the relative hydrophobicity and hydrophilicity of each surface.

### 2.4. Self-Cleaning of Tri-Layered Scaffold

To assess the self-cleaning efficacy of the electrospun EPS/PTFE-µPs/SiO_2_-NPs nanofibrous TSL surface to eliminate contaminated particles, Fe_2_O_3_-NPs were dispersed onto the nanofibrous layer surface, and then rapidly flowing water droplets were applied using a syringe. This assessment of the surface’s ability to effectively cleanse itself by eliminating the Fe_2_O_3_-NPs highlighted the self-cleaning potential inherent in the electrospun EPS/PTFE-µPs/SiO_2_-NPs nanofibrous TSL structure.

### 2.5. Antibacterial Study of Tri-Layered Scaffold

The bacterial viability counting approach was used to evaluate the antibacterial capability of the electrospun MFL/HAp/Ag-NPs nanofibrous BHL of the tri-layered scaffold against Gram-negative (*E. coli*) and Gram-positive (*S. aureus*) bacteria as experimental strains. *E. coli* and *S. aureus* were inoculated in a slant culture medium and cultured at 37 °C for 44 h. To compare the antibacterial activities against *E. coli* and *S. aureus*, BHL of the tri-layered scaffold with different weight percentages of Ag-NPs (0.0, 0.1, 0.4, 0.8, and 1.5 wt%) were cut and added to each broth with a density of 2.5 × 10^4^ bacteria/mL, and the broths were incubated at 37 °C for 24 h for the bacteria to culture. An amount of 100 µL bacteria were spread on Petri dishes and then incubated at 37 °C for 24 h to allow the cells to culture. The number of colonies on different Petri dishes was calculated. The bacterial inhibition efficiency was calculated using the following Equation (1):

(1) $$\mathrm{Bactericidal}\;\mathrm{Inhibition}\;\mathrm{Efficiency}=\frac{A-B}{A}\times 100\%$$

where *A* is the number of bacteria in the broth treated with the BHL without Ag-NPs (0.0 wt%) after 24 h of contact time, and *B* is the number of bacteria in the broth treated with the BHL with Ag-NPs after 24 h of contact time. The BHL without Ag-NPs (0.0 wt%) was used as a negative control group for testing the antibacterial activity of other BHLs with different weight percentages of Ag-NPs (0.1, 0.4, 0.8, and 1.5 wt%).

### 2.6. In Vitro Cytotoxicity Study of Tri-Layered Scaffold

**Figure 2:**
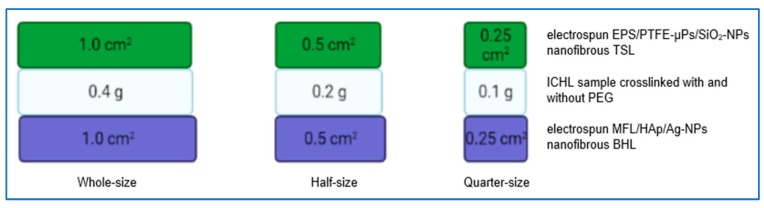
Comparison of different sizes of tri-layered scaffolds on promoting cell growth.

The in vitro cytotoxicity of the fabricated tri-layered scaffold was assessed by using a 3-(4,5-dimethylthiazol-2-yl)-2,5-diphenyl tetrazolium bromide (MTT) assay. Fibroblast cell lines (3T3 and L-929 cells) were obtained from ATCC. The cells were seeded at a density of 2 × 10^4^ cells per well in 24-well plates with DMEM culture medium supplemented with 10% fetal bovine serum (FBS), 100U/mL Penicillin, and 0.1mg/mL streptomycin at 37 °C in a 5% CO_2_ incubator. After 24 h of cell culturing, each ultraviolet (UV)-irradiated tri-layered scaffold of different sizes (0.25, 0.5, and 1.0 cm^2^) was immersed in each cell-containing well to co-culture for 4 days at 37 °C in a 5% CO_2_ incubator. Each tri-layered scaffold consisted of the TSL with EPS, 10 wt% PTFE-µPs and SiO_2_-NPs inclusions, ICHL produced from CS, other bioactive contents (BSA and aloe vera) with and without PEG, and the BHL with MFL, HAp, and 1.5 wt% Ag-NPs. The ICHL samples of different masses (0.1, 0.2, and 0.4 g) were sandwiched between the TSL and BHL, as shown in Figure 2. Table 1 summarizes the scale-size effects of all three layers of the tri-layered scaffold. After four days of incubation, the tri-layered scaffold samples were removed, and the culture medium was discarded. Subsequently, 500 µL of fresh culture medium was inserted into each well along with 50 µL MTT solution, followed by incubation at 37 °C for 4 h. The media was then removed, and 500 µL of sodium dodecyl sulfate-hydrochloric acid (SDS-HCl) solution was added to each well and incubated at 37 °C for 2 h to dissolve the formazan crystals. Then, 200 µL of the resulting solution was aliquoted into each well of a 96-well plate, and the absorbance was measured at 570 nm using an ELISA reader. For comparative analysis, a control group was included using a culture medium without any extracts.

### 2.7. Statistical Analysis

Data were expressed as mean values ± standard deviation (SD). Statistical analysis among groups were performed using SPSS (IBM, Chicago, IL, USA) by single factor analysis of variance test (one-way ANOVA) with the LSD formula for post hoc multiple comparisons, or *T*-test for two groups. *p* value less than 0.05 is considered significant difference.

## 3. Results and Discussion

### 3.1. Morphology

**Figure 3:**
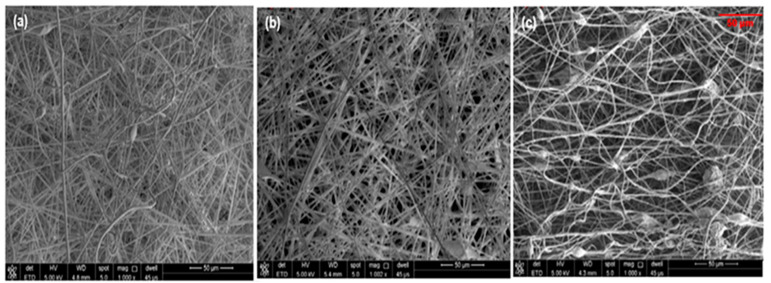
SEM micrographs of electrospun EPS/PTFE-µPs/SiO_2_-NPs nanofibrous TSL with: (**a**) EPS with 5 wt% PTFE-µPs and SiO_2_-NPs inclusions; (**b**) EPS with 10 wt% PTFE-µPs and SiO_2_-NPs inclusions; and (**c**) EPS with 15 wt% PTFE-µPs and SiO_2_-NPs inclusions.

SEM images were obtained from four locations on each layer of the tri-layered scaffold to investigate surface morphology. Figure 3 shows these morphologies of the electrospun EPS/PTFE-µPs/SiO_2_-NPs nanofibrous TSLs with different weight percentages of PTFE-µPs and SiO_2_-NPs inclusions. Each TSL exhibited distinct aligned surface morphologies. As the PTFE-µPs and SiO_2_-NPs inclusions increased to 15 wt%, the nanofibrous matrix formed sphere-shaped beads on the fibrous surface, and the surface roughness increased. Moreover, the combination of both PTFE-µPs and SiO_2_-NPs in the polymeric solution had an increased viscosity. Consequently, the charging jet did not break down into smaller units as a result of chain entanglements [25]. When the viscosity of the combination of both PTFE-µPs and SiO_2_-NPs in the polymeric solution is increased, the polymer chains experience more resistance to flow. This leads to higher Coulombic stress because the charged segments of the polymer chains repel each other more strongly due to the slower flow. As a result, the charged jet elongates further, creating even thinner fibers [26]. Additionally, the hierarchical micro/nanostructures formed due to the synergistic effect of the PTFE-µPs and SiO_2_-NPs inclusions on the fibrous surface provided enough roughness to achieve super hydrophobicity [27].

**Figure 4:**
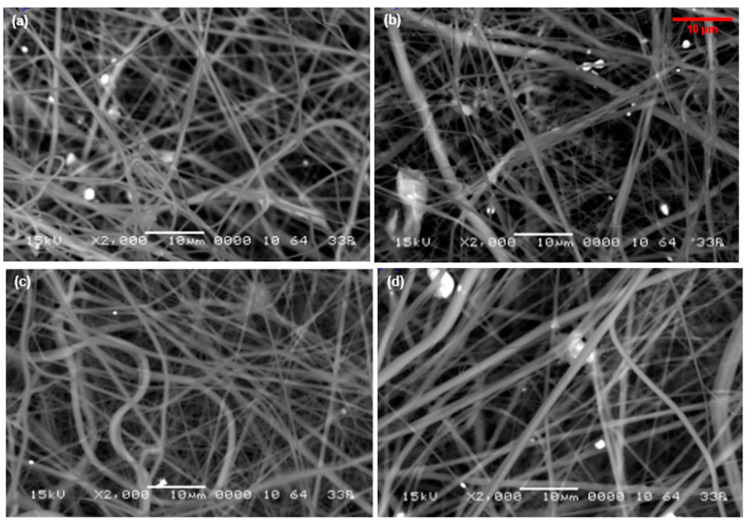
SEM micrographs of electrospun MFL/HAp/Ag-NPs nanofibrous BHL with: (**a**) MFL with HAp and 0.1 wt% Ag-NPs; (**b**) MFL with HAp and 0.4 wt% Ag-NPs; (**c**) MFL with HAp and 0.8 wt% Ag-NPs; and (**d**) MFL with HAp and 1.5 wt% Ag-NPs.

Figure 4 shows SEM images of the surfaces of electrospun MFL/HAp/Ag-NPs nanofibrous BHLs with HAp and different weight percentages of Ag-NPs. Upon visualization, the BHLs exhibit irregular and random orientations with different surface roughness and some sphere-shaped beaded surface structures. However, the SEM image of electrospun MFL/HAp/Ag-NPs nanofibrous BHL with 1.5 wt% Ag-NPs exhibits a stable and aligned structure along one direction, as shown in Figure 4d. Also, Figure 4d illustrates growth in the average fiber diameter of the surface structure, which corresponds to the rise in viscosity of the polymeric solution as the inclusion of HAp and Ag-NPs increased [28,29]. This finding suggests that incorporating HAp and Ag-NPs plays a crucial role in modifying the behavior of the polymeric solution during the electrospinning process, ultimately influencing the properties of the resulting fibrous layer [30], including HAp and Ag-NPs’ increased surface roughness, potentially improving the hydrophilicity of the BHL. Also, the surface roughness may promote cell adhesion and proliferation [31], as shown in MTT assays (section below). Additionally, the Ag-NPs bound to the fibrous surface structures may improve the antimicrobial properties of the electrospun MFL/HAp/Ag-NPs nanofibrous BHLs, as the Ag-NPs create inhibition sectors against microbes. The electrospun MFL/HAp/Ag-NPs nanofibrous BHLs were designed to replicate the characteristics of the ECM by utilizing components with dimensions on the nanometer and micrometer scale. All electrospun MFL/HAp/Ag-NPs nanofibrous BHLs were within the nanometer range, offering favorable conditions for facilitating cellular growth and bioactivity [32].

**Figure 5:**
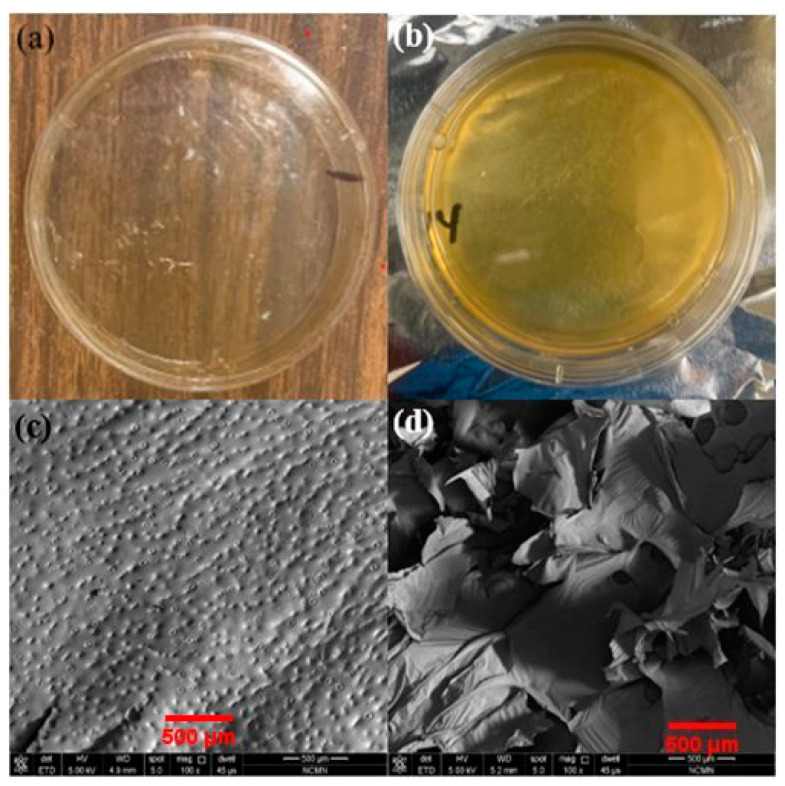
Upper panels: Petri dishes of ICHL samples (**a**) without PEG and (**b**) cross-linked with PEG. Lower panels: SEM micrographs of ICHL samples (**c**) without PEG and (**d**) cross-linked with PEG.

Surface morphology is also a vital parameter for ICHL characterization because it affects cellular and wound healing behavior. Figure 5a,b illustrates the ICHL sample without PEG and the other ICHL sample cross-linked with PEG in separate Petri dishes, respectively. Figure 5c exhibits an SEM image of the surface of the ICHL sample without PEG. Notably, no significant morphology change was observed, which can be attributed to the absence of the PEG concentration in this formulation. Figure 5d displays an SEM image of the surface of the ICHL sample cross-linked with PEG, which revealed the impact of cross-linking on the formation of CS and PEG bonds. These cross-linked networks are immensely important in controlled drug/antibiotic delivery applications [33,34]. Consequently, the ICHL sample cross-linked with PEG has a beneficial influence on chronic wound healing due to its porous nature [35]. In summary, the SEM images show significant differences in the morphology of the ICHL sample cross-linked with and without PEG, as the micrographs are distinguishable.

### 3.2. Surface Chemistry Analysis

**Figure 6:**
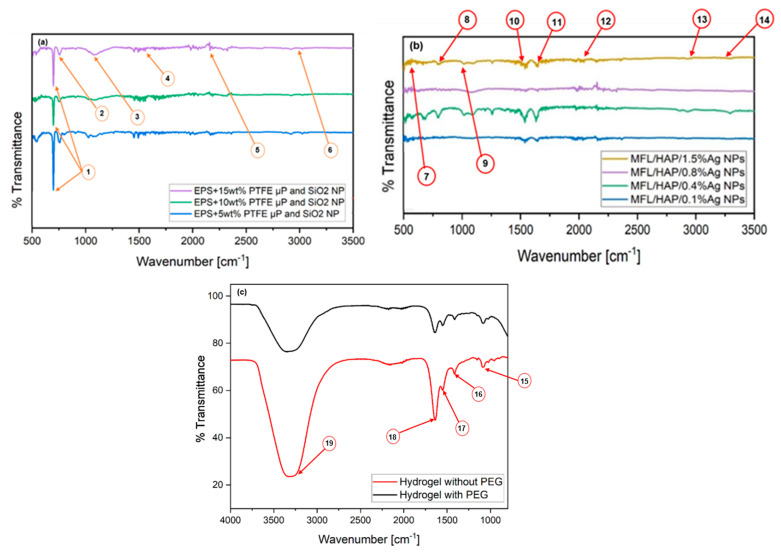
FTIR spectra showing comparison of three distinct layers of tri-layered scaffold: (**a**) electrospun EPS/PTFE-µPs/SiO_2_-NPs nanofibrous TSLs with different weight % of PTFE-µPs and SiO_2_-NPs inclusions; (**b**) electrospun MFL/HAp/Ag-NPs nanofibrous BHLs with HAp and different weight % of Ag-NPs; and (**c**) ICHL samples cross-linked with and without PEG. Arrows correspond to peaks detailed in Table 2.

The FTIR spectroscopy analysis provides valuable information on the surface chemistry of each layer of the tri-layered scaffold, indicating the presence of various chemical bonds and functional groups. The FTIR spectra of the electrospun EPS/PTFE-µPs/SiO_2_-NPs nanofibrous TSLs are presented in Figure 6a. The analysis reveals that the TSL with different weight percentages of PTFE-µPs and SiO_2_-NPs inclusions have more absorption peaks and are nearly identical despite the various weight percentages. The peaks observed in the spectra represent the different vibrational modes of the atoms within the TSL. The TSL with 5 wt% PTFE-µPs and SiO_2_-NPs inclusions shows a strong peak at 698.04 cm^−1^, which is attributed to the presence of out-of-phase ring deformation of monosubstituted aromatic rings. The intensity of this peak weakens as the weight percentages of PTFE-µPs and SiO_2_-NPs inclusions increase. For both TSLs with 10 and 15 wt% PTFE-µPs and SiO_2_-NPs inclusions, observed peaks at 757.49 cm^−1^ and 1097.08 cm^−1^ correspond to the strong C–Cl stretching and strong C–O stretching of a secondary alcohol group. Peaks observed at approximately 1652.92 cm^−1^ and 2190.10 cm^−1^ indicate the medium C=C stretching of the vinylidene group and weak C≡C stretching of the alkyne group, respectively. The tiny peak observed at 2923.60 cm^−1^ corresponds to the medium C–H stretching of the alkane group. These peaks are further supported by findings in the literature [28,36,37]. Upon analyzing the observed peaks, it can be inferred that incorporating PTFE-µPs and SiO_2_-NPs into the EPS polymeric solution increased the intensity and number of absorption peaks. This observation suggests the presence of additional functional groups that may have formed during the various preparation stages, including stirring, sonication, or electrospinning of the EPS polymeric solution. These additional functional groups are believed to result from chemical reactions or bonding processes within the solution [38].

The FTIR spectra of the electrospun MFL/HAp/Ag-NPs nanofibrous BHLs with HAp and different weight percentages of Ag-NPs are depicted in Figure 6b. It is worth noting that the FTIR spectra of the BHLs with varying weight percentages of Ag-NPs are nearly identical, except for the BHL with 0.4 wt% Ag-NPs. These spectra show the strong and sharp absorption peaks of the strong N-O stretching of the nitro compound group at 1540.83 cm^−1^ and the medium C=C stretching of the conjugated alkenes group observed at 1646.85 cm^−1^. As evident in Figure 6b, there are no significant differences between the spectra of all other electrospun MFL/HAp/Ag-NPs nanofibrous BHLs. However, a few other noticeable peaks are observed on all the electrospun MFL/HAp/Ag-NPs nanofibrous BHLs with 0.1, 0.4, 0.8, and 1.5 wt% Ag-NPs. These peaks observed at 532.80, 752.40, 1083.92, 2034.86, 2900.30, and 3298.06 cm^−1^ correspond to the strong C–Br stretching of the halo compound group, strong C–H bending of the monosubstituted compound group, strong C–O stretching of the primary alcohol group, strong N=C=S stretching of the isothiocyanate group, medium C–H stretching of the alkane group, and strong and broad O–H stretching of the carboxylic acid group in its structure, respectively [39].

FTIR spectroscopy is also a helpful characterization technique to gain insight into the chemical functional groups of ICHL conformations. FTIR spectra were obtained for ICHL samples cross-linked with and without PEG, as presented in Figure 6c. The ICHL samples cross-linked with PEG displayed a strong curve absorption peak at 3351.24 cm^−1^, indicating the formation of CS and PEG bonds. This peak is characteristic of the strong O–H stretching group in CS-PEG cross-linking, thus confirming the existence of cross-linking in CS. Cross-linking effectively prevented undesirable water absorption and retention by ICHL, enhancing the tri-layered scaffold’s overall durability [40]. Additional absorption peaks are observed at 1091.65, 1415.88, 1557.51, and 1644.97 cm^−1^, which are attributed to strong C–O stretching of the secondary alcohol group, strong S=O stretching of the sulfate group, strong N–O stretching of the nitro compound group, and medium C=C stretching of the cyclic alkene group, respectively. Similarly, the ICHL samples without PEG showed a strong curve absorption peak at 3320.39 cm^−1^, corresponding to the strong O–H stretching group. Also, the ICHL sample without PEG showed several absorption peaks at 1091.90, 1414.75, 1551.54, and 1636.53 cm^−1^. These peaks correspond to the strong C–O stretching of the secondary alcohol group, strong S=O stretching of the sulfate group, strong N–O stretching of the nitro compound group, and medium C=C stretching of the cyclic alkene group, respectively. These FTIR results show no significant differences between the spectra of both ICHL conformations cross-linked with and without PEG because the absorption peaks observed were identical and no new peaks were noticed [41,42]. However, the ICHL samples lacking PEG possess absorption peaks slightly sharper and stronger than those with PEG, which suggests that cross-linking PEG into the ICHL conformation reduces the strength of the absorption peaks and prevents unwanted water absorption, as depicted in Figure 6c.

### 3.3. Thermal Degradation

Thermogravimetric analysis was utilized as the characterization technique to investigate the thermal degradation behavior of each layer of the tri-layered scaffold at elevated temperatures. Derivative Thermogravimetry (DTG) analysis was used to determine the temperature at which the material loss was the most.

**Figure 7:**
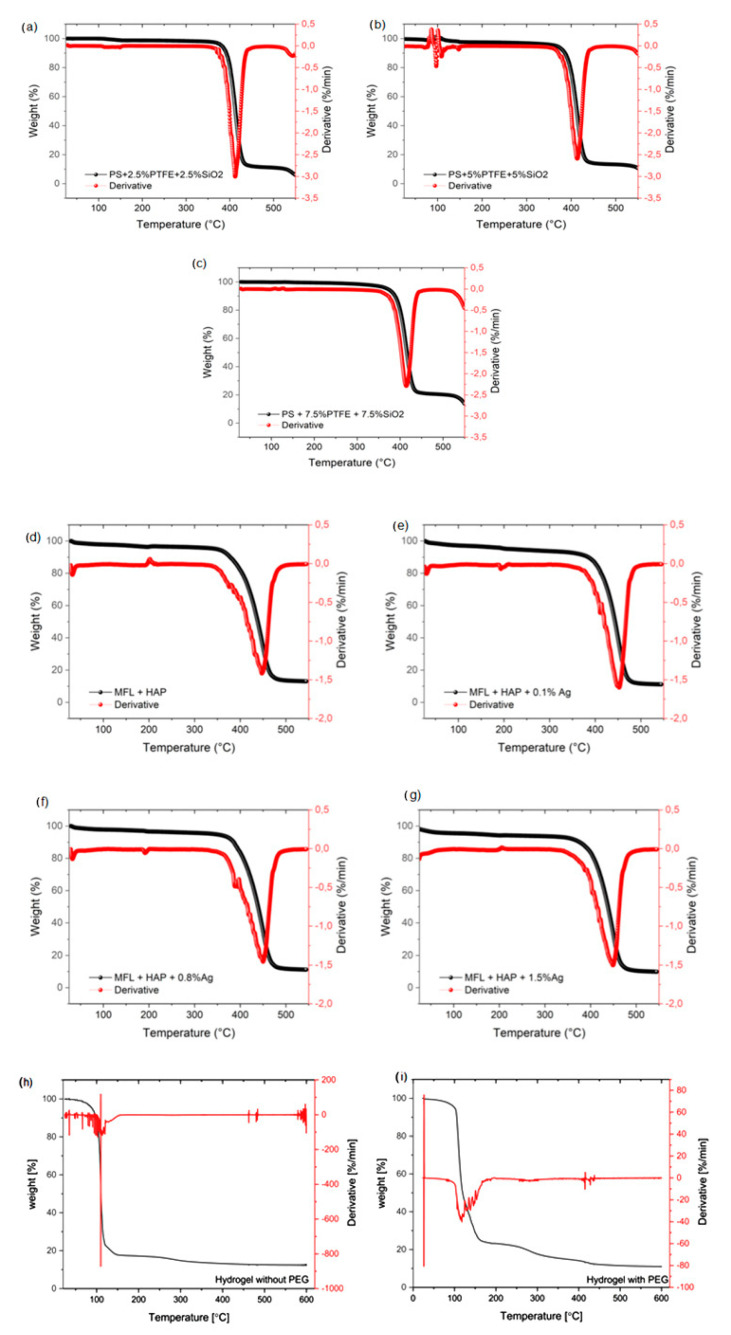
TGA-DTG profiles of three distinct layers of tri-layered scaffold: (**a**–**c**) electrospun EPS/PTFE-µPs/SiO_2_-NPs nanofibrous TSLs with different weight percentages of PTFE-µPs and SiO_2_-NPs inclusions; (**d**–**g**) electrospun MFL/HAp/Ag-NPs nanofibrous BHLs with HAp and different weight percentages of Ag-NPs; and (**h**,**i**) ICHL samples cross-linked with and without PEG.

TGA/DTG profiles of electrospun EPS/PTFE-µPs/SiO_2_-NPs nanofibrous TSLs, MFL/HAP/Ag nanofibrous BHLs, and ICHL samples cross-linked with and without PEG were performed under a nitrogen atmosphere to determine the effect of the polymers’ content on the thermal stability of the tri-layered scaffold. TGA profiles of TSLs with varying weight percentages of PTFE-µPs and SiO_2_-NPs inclusions were compared, as depicted in Figure 7a–c. The obtained thermograph profiles provide information about the thermal stability, decomposition temperature, and weight loss of the TSLs as they are heated. The degradation mechanism of TSL involves intermolecular and intramolecular transfer reactions, depolymerization, and random scission. These processes lead to a decrease in the molecular weight of the TSLs [43]. Figure 7a–c demonstrates that the degradation of the electrospun EPS/PTFE/SiO_2_ nanofibrous TSLs occurs in three stages. Each TSL displays a unique weight loss profile at various temperatures. Interestingly, the TSL with 2.5 wt% PTFE-µPs and 2.5 wt% SiO_2_-NPs inclusions, and 7.5 wt% PTFE-µPs and 7.5 wt% SiO_2_-NPs inclusions exhibit identical weight loss profiles from 0 °C to 400 °C. However, the TSL with 2.5 wt% PTFE-µPs and 2.5 wt% SiO_2_-NPs inclusions exhibit approximately 7% weight loss and lower thermal stability than the other electrospun EPS/PTFE/SiO_2_ nanofibrous TSLs at these temperatures, as depicted in Figure 7a–c. This observed effect was not amplified by the varying weight percentages of PTFE-µPs and SiO_2_-NPs inclusions in this study. The first stage of weight loss was due to the evaporation of the trapped solvent (DMF and C_3_H_6_O) and the loss of absorbed moisture. This stage was observed consistently in all electrospun EPS/PTFE/SiO_2_ nanofibrous TSLs [44].

The second stage of weight loss for all the electrospun EPS/PTFE/SiO_2_ nanofibrous TSLs observed a notable decrease in thermal stability from 400 °C to 445 °C, coinciding with the degradation of EPS caused by decomposition that took place on the polymer side chain (T-ds). The decomposition temperature of the polymer side chain was around 360 °C. The noticeable decrease in thermal stability during this stage highlights the susceptibility of EPS to degradation, emphasizing the importance of understanding its thermal properties for effective scaffold design. It can be observed that there is a significant overlap in the TGA profiles of all electrospun EPS/PTFE/SiO_2_ nanofibrous TSLs from 400 °C to 445 °C, which indicates that they exhibit a similar weight loss profile during this temperature range. Furthermore, incorporating micro- and nano-inclusions in the TSLs amplifies char residue as the temperature increases. This observation highlights the influence of micro- and nano-inclusions on the thermal behavior of the TSLs because they enhance their ability to withstand higher temperatures before undergoing subsequent decomposition. The polymer residues undergo further decomposition at approximately 445 °C, representing the third stage of degradation. The observed weight loss within this temperature range demonstrates that the electrospun EPS/PTFE/SiO_2_ nanofibrous TSLs undergo various degradation levels, resulting in the generation of char residue, attributed to the degradation of the main chain (T-dm) of PTFE, thereby signifying its role in the overall degradation process [45,46].

Figure 7d–g shows TGA profiles of electrospun MFL/HAp/Ag-NPs nanofibrous BHLs with HAp and various weight percentages of Ag-NPs. The degradation mechanism of the BHL includes intermolecular and intramolecular transfer reactions, depolymerization, and random scission, reducing the molecular weight of the BHLs. This reduction is attributed to the breaking of chemical bonds between molecules within the BHLs [43]. Similarly, it appears that each electrospun MFL/HAp/Ag-NPs nanofibrous BHL undergoes three distinct degradation stages. In addition, the weight loss profile of each BHL varies at different temperatures, depending on the presence of inclusions. The initial stage of degradation occurs within a temperature range of 0 °C to 375 °C. At this stage, most of the electrospun MFL/HAp/Ag-NPs nanofibrous BHLs demonstrate excellent thermal stability, except for BHLs with 0.4 and 1.5 wt% Ag-NPs. Electrospun MFL/HAp/Ag-NPs nanofibrous BHL with 1.5 wt% Ag-NPs suffer a significant weight loss of 7% at an elevated temperature, displaying the worst thermal stability compared to all other BHLs. Furthermore, the TGA profiles illustrate that increasing the weight percentage of Ag-NPs above 0.1 wt% does not enhance thermal stability from 0 °C to 375 °C. Thus, it was determined that 0.1 wt% Ag-NPs was the ideal weight percentage of Ag-NPs to achieve the best thermal stability within this temperature range. The first stage of weight loss occurred from 0 °C to 375 °C due to the evaporation of the trapped solvent (HCOOH and CH_3_COOH) and the catastrophic effect of the absorbed moisture [47].

The second stage of degradation occurs from 375 °C to 460 °C with further weight loss of electrospun MFL/HAp/Ag-NPs nanofibrous BHLs, which is linked to the degradation of the MFL and the side chain of HAp (T-ds). During the second stage of degradation, BHLs with 0.4 and 1.5 wt% Ag-NPs exhibit excellent thermal stability compared to other BHLs. The enhanced thermal stability observed in the second stage of degradation could be due to the formation of a protective layer of Ag-NPs on the surface of the BHLs, as illustrated in Figure 4. This protective layer shields the electrospun MFL/HAp/Ag-NPs nanofibrous BHLs from degradation at elevated temperatures [27]. Furthermore, incorporating HAp and Ag-NPs in the BHLs contributes to the enhancement of molecular interactions and impedes the mobility of polymeric chains. Also, it elevates the energy requirement for the movement and fracture of the polymeric chains [48,49]. In the third stage of degradation, the HAp and MFL residues continue to degrade at close to 470 °C, reflecting the breakdown of the HAp chain (T-dm). Additionally, it was observed that electrospun MFL/HAp nanofibrous BHLs (without Ag-NPs) suffer degradation at lower temperatures compared to all other electrospun MFL/HAp/Ag-NPs nanofibrous BHLs with Ag-NPs.

Similarly, TGA was utilized to study and measure the thermal breakdown of the fabricated ICHL conformations at high temperatures. Figure 7h and i illustrates the TGA profiles of these ICHL samples, which are compared with and without PEG inclusions. The degradation process of the ICHL samples leads to a decline in their molecular weight. The data shows that both ICHL samples undergo three stages of degradation, indicating that each ICHL sample has a distinct weight-loss profile at different temperatures, regardless of the cross-linking of CS and PEG bonds (Figure 7h,i). The initial stage of degradation occurs from 0 °C to 100 °C, indicated as a minor decline in weight for both ICHL samples, which is attributed to solvent evaporation trapped within the samples during fabrication. Hence, they display good thermal stability during the initial stage of degradation. Afterward, the second stage of degradation occurs from 100 °C to 155 °C, indicated as a considerable weight loss in both ICHL conformations. However, ICHL samples cross-linked with PEG display better thermal stability during this phase. ICHL samples cross-linked with PEG and without PEG exhibit weight losses of about 65% and 73%, respectively. The weight loss during the second stage of degradation is attributed to the CS and PEG side chain degradation. The third stage of degradation occurs in the temperature range from 155 °C to 300 °C. Both ICHL samples exhibit an identical weight loss profile, indicating that the main chain of CS and PEG is being degraded. At this stage, only a minimal amount of weight loss is observed, which implies that the ICHL conformations have good thermal stability. Here, DTG results agree well with the TGA results for all the samples.

### 3.4. Water Contact Angle and Wettability

**Figure 8:**
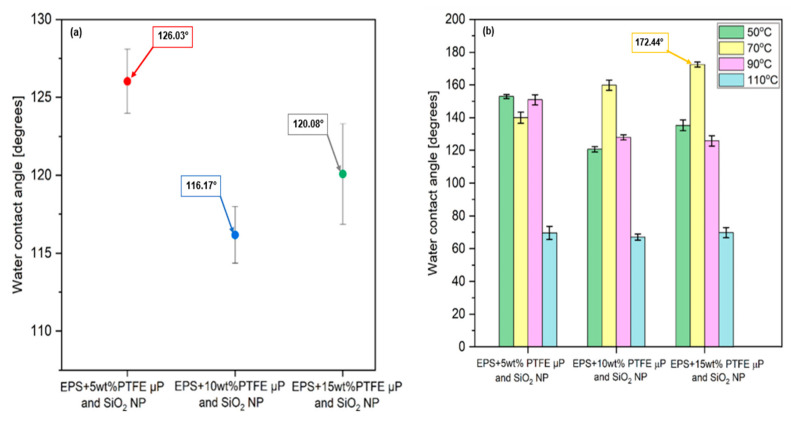
(**a**) WCAs of electrospun EPS/PTFE-µPs/SiO_2_-NPs nanofibrous TSLs with different weight percentages of PTFE-µPs and SiO_2_-NPs inclusions under no heat treatment condition; (**b**) effect of heat treatment on WCAs of electrospun EPS/PTFE-µPs/SiO_2_-NPs nanofibrous TSLs with different weight percentages of PTFE-µPs and SiO_2_-NPs inclusions.

Next, we assessed the surface characteristics of theTSL and BHL of the tri-layered scaffold in terms of wettability using water contact angle measurements at room temperature. The electrospun EPS/PTFE-µPs/SiO_2_-NPs nanofibrous TSLs exhibited an elevated level of hydrophobicity and very low liquid/solid interface adhesion. Increasing surface roughness and decreasing surface energy through surface modification is necessary to form micro/nano-scale surface structures to create a superhydrophobic and self-cleaning surface layer [50,51]. Wettability of the electrospun EPS/PTFE-µPs/SiO_2_-NPs nanofibrous TSLs is defined by their WCAs, which measure how easily water droplets spread on the layer surface. The data presented in Figure 8a illustrate that introducing PTFE-µPs and SiO_2_-NPs inclusions into the polymeric solution increased the WCAs of electrospun EPS/PTFE-µPs/SiO_2_-NPs nanofibrous TSLs. The TSL with 5 wt% PTFE-µPs and SiO_2_-NPs inclusions resulted in the maximum WCA of 126.03°. This increase in WCA is attributed to the enhanced surface roughness and agglomeration, as illustrated in Figure 3a, which visually depicts the surface morphology of the electrospun EPS/PTFE-µPs/SiO_2_-NPs nanofibrous TSL after incorporating micro- and nanoinclusions. The increase in weight percentages of PTFE-µPs and SiO_2_-NPs inclusions to 10 and 15 wt% decreases the WCAs of the TSLs to 116.17° and 120.08°, respectively. It is hypothesized that the wettability of the electrospun EPS/PTFE-µPs/SiO_2_-NPs nanofibrous TSLs could be exploited through heat treatment. To test this hypothesis, the electrospun EPS/PTFE-µPs/SiO_2_-NPs nanofibrous TSLs were heat treated in an electric oven at various temperatures (50 °C, 70 °C, 90 °C, and 110 °C) for 6 h. Results of the change in (super)hydrophobicity are illustrated in Figure 8b. It is possible to manipulate the electrospun nanofibrous layer’s surface energy and create a surface with either high or low wettability by varying the heat treatment temperature.

The level of hydrophobicity of the electrospun EPS/PTFE-µPs/SiO_2_-NPs nanofibrous TSL improves steadily as the heat treatment temperature is elevated from 50 °C to 90 °C. However, a decline in hydrophobicity is observed when the heat treatment temperature increases to 110 °C, making the TSL more hydrophilic. Therefore, the heat treatment was maintained at 70 °C for 6 h. The highest WCAs were recorded for electrospun EPS/PTFE-µPs/SiO_2_-NPs nanofibrous TSLs with 10 and 15 wt% PTFE-µPs and SiO_2_-NPs inclusions as 172.44° and 159.82°, respectively. On the other hand, the TSL with 5 wt% PTFE-µPs and SiO_2_-NPs inclusions had the highest WCA of 152.95° at 50 °C. The WCA results show that the optimal condition for achieving the highest WCA was when the electrospun EPS/PTFE-µPs/SiO_2_-NPs nanofibrous TSL with 10 wt% PTFE-µPs and SiO_2_-NPs inclusions were heat treated at 70 °C for 6 h. The higher the WCA, the higher the greater the superhydrophobicity. The WCA for the heat-treated electrospun EPS/PTFE-µPs/SiO_2_-NPs nanofibrous TSL with 10 wt% PTFE-µPs and SiO_2_-NPs inclusions was measured to be 172.44°, which marked it as superhydrophobic. The enhanced hydrophobicity in the heat-treated electrospun EPS/PTFE-µPs/SiO_2_-NPs nanofibrous TSL was attributed to a possible reduction in the number of functional groups on the nanofibrous layer surface. This reduction in functional groups decreased the polar structures interacting with water molecules. Additionally, heat treatment could cause a reorientation of the micro- and nanoinclusions on the TSLs, causing an increase in the surface roughness [52,53].

The WCA results obtained in this study are much better than in other studies using similar materials. Liang et al. fabricated waterproof-breathable nanofiber membranes with superhydrophobic and self-cleaning properties. These membranes were composed of PTFE-µPs/SiO_2_-NPs and exhibited a bead-like structure. The prepared membranes displayed remarkable superhydrophobicity, as indicated by a WCA measurement of 155° [54]. In a separate research study by Chen et al., a bioinspired superhydrophobic surface was developed using a hierarchically wrinkled nanoporous polymer, which involved coating a polystyrene (PS) sheet with a PTFE emulsion and subjecting it to thermal treatment to eliminate surfactants that existed around the PTFE-µPs. This thermal contraction process induced wrinkling on the PTFE surfaces, which exhibited a WCA of approximately 167° [55].

**Figure 9:**
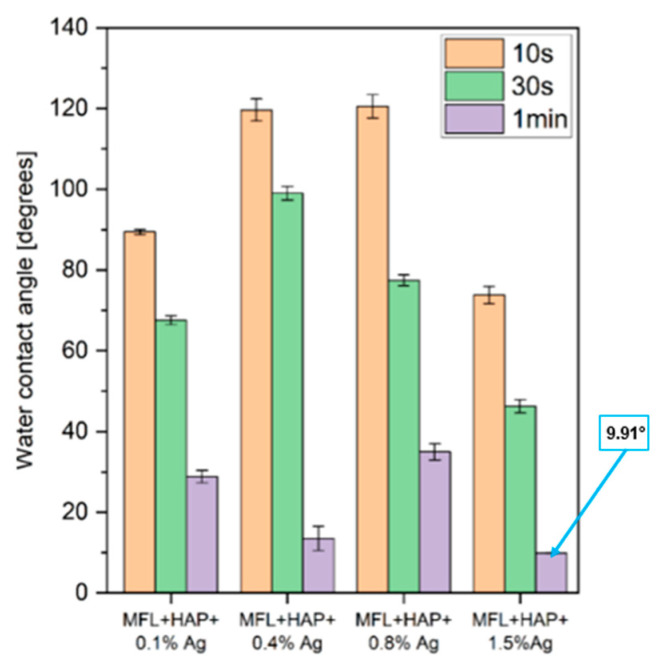
Wettability transition of electrospun MFL/HAp/Ag-NPs nanofibrous BHLs with HAp and different weight percentages of Ag-NPs for 10 s, 30 s, and 60 s.

Subsequently, the wettability of the electrospun MFL/HAp/Ag-NPs nanofibrous BHLs with 0.1, 0.4, 0.8, and 1.5 wt% of Ag-NPs was assessed by WCA and measured at different time intervals of 10 s, 30 s, and 60 s, as depicted in Figure 9. High WCAs can be seen for the electrospun MFL/HAp/Ag-NPs nanofibrous BHLs with 0.4 and 0.8 wt% Ag-NPs. Both BHLs had WCAs of 119.61° and 120.54°, respectively, in the first 10 s after water drops. However, after that period, a gradual transition to hydrophilicity occurred for those BHLs in 60 s and 30 s, respectively. It was observed that the WCAs significantly decreased within a 60 s interval for all electrospun MFL/HAp/Ag-NPs nanofibrous BHLs, which indicated that the BHLs experienced a transition from being hydrophobic to hydrophilic during this interval. The inclusion of HAp and various weight percentages of Ag-NPs into the polymeric solution improved the hydrophilicity of the BHLs (Figure 9), which was deduced from the fact that the WCAs of the BHLs with inclusions were lower after 60 s of water droplet release. The electrospun MFL/HAp/Ag-NPs nanofibrous BHL with 1.5 wt% Ag-NPs displayed the most hydrophilic behavior because the WCAs were instantly below 90° when water droplets were released onto their surfaces. The average WCA of the electrospun MFL/HAp/Ag-NPs nanofibrous BHL with 1.5 wt% Ag-NPs was 9.91°, suggesting hydrophilicity.

These findings reveal that the wettability characteristics of the TSL and BHL exhibit distinct variations, indicating a unidirectional change in hydrophobicity across the layers of the tri-layered scaffold. Specifically, the TSL demonstrates the highest level of hydrophobicity, reaching a superhydrophobic state, whereas the BHL exhibits a significantly higher degree of hydrophilicity. This arrangement of layers within the tri-layered scaffold is crucial to establish a moisture content gradient that mimics skin. The top layer of skin, the epidermis, possesses an inherently hydrophobic nature, which plays a vital role in preventing excessive absorption of external fluids and acts as a barrier against the infiltration of foreign particles into the body. The top layer of the tri-layered scaffold was engineered to exhibit (super)hydrophobic properties, thereby effectively protecting the wound against potential infections caused by foreign elements and fulfilling a similar function. The WCA for the heat-treated electrospun EPS/PTFE-µPs/SiO_2_-NPs nanofibrous TSL with 10 wt% PTFE-µPs and SiO_2_-NPs inclusions was 172.44°, firmly establishing it as superhydrophobic. The bottom layer of the tri-layered scaffold has been specifically engineered to exhibit a hydrophilic nature, targeting the facilitation of efficient attachment and integration at the wound site. To achieve this, the average WCA of electrospun MFL/HAp/Ag-NPs nanofibrous BHL with 1.5 wt% Ag-NPs was 9.91°, indicating its hydrophilic nature. Furthermore, this layer would not only retain moisture but also facilitate the movement of macromolecules, thereby enabling effective fluid exchange. This attribute holds significant value for chronic wound healing and research on regenerative medicine.

### 3.5. Self-Cleaning Study

**Figure 10:**
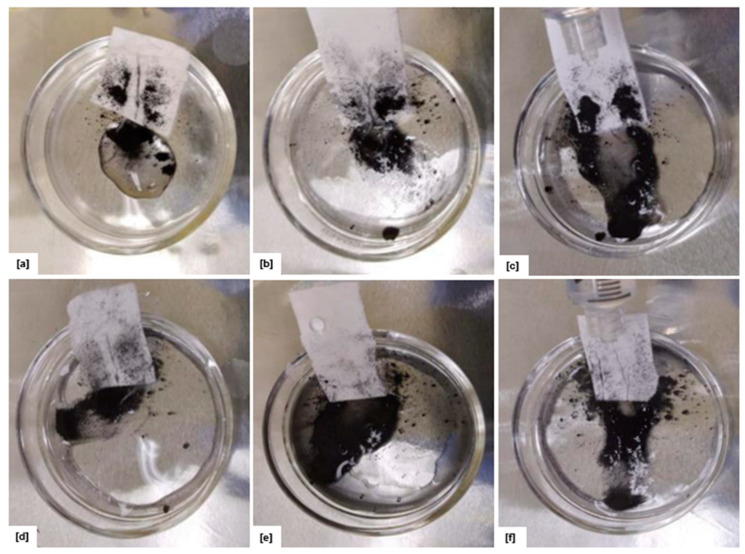
Self-cleaning test of electrospun EPS/PTFE-µPs/SiO_2_-NPs nanofibrous TSL with: (**a**) EPS with 5 wt% PTFE-µPs and SiO_2_-NPs inclusions; (**b**) EPS with 10 wt% PTFE-µPs and SiO_2_-NPs inclusions; (**c**) EPS with 15 wt% PTFE-µPs and SiO_2_-NPs inclusions; (**d**) heat-treated EPS with 5 wt% PTFE-µPs and SiO_2_-NPs inclusions at 70 °C; (**e**) heat-treated EPS with 10 wt% PTFE-µPs and SiO_2_-NPs inclusions at 70 °C; and (**f**) heat-treated EPS with 15 wt% PTFE-µPs and SiO_2_-NPs inclusions at 70 °C.

A comprehensive investigation was conducted to assess the self-cleaning capability of the electrospun EPS/PTFE-µPs/SiO_2_-NPs nanofibrous TSL, characterized by its unique property of repelling water. This unique characteristic enables water droplets to effectively cleanse the surface by eliminating contaminants upon release. A syringe was used to release distilled water droplets onto the TSL surface, covered with Fe_3_O_4_ powder, to evaluate this self-cleaning performance. The setup thoroughly examined the TSL efficacy in effectively eliminating contaminants and sustaining its self-cleaning functionality. As depicted in Figure 10, all electrospun EPS/PTFE-µPs/SiO_2_-NPs nanofibrous TSLs were positioned at a fixed tilt angle of 45°. The objective was to investigate the influence of micro- and nanoinclusions and heat treatment on the self-cleaning capability of the TSL.

**Figure 11:**
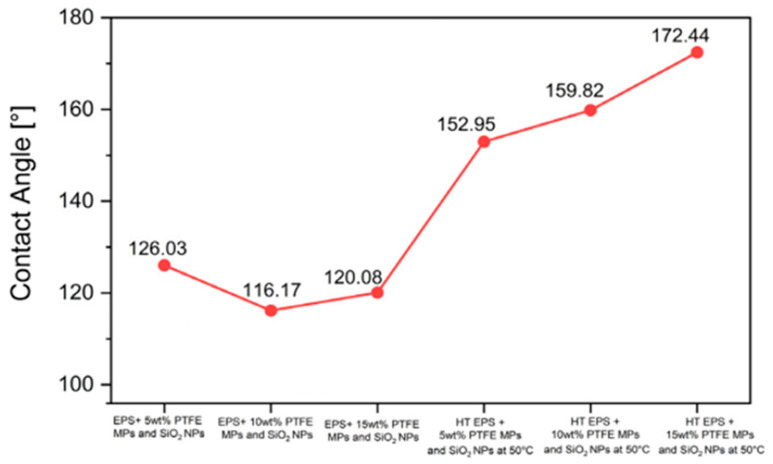
Graph showing WCAs after self-cleaning test of electrospun EPS/PTFE-µPs/SiO_2_-NPs nanofibrous TSLs with different weight percentages of PTFE-µPs and SiO_2_-NPs inclusions under no heat treatment and heat treatment condition at 70 °C.

It was observed that the electrospun EPS/PTFE-µPs/SiO_2_-NPs nanofibrous TSL with 5 wt% PTFE-µPs and SiO_2_-NPs inclusions exhibited exceptional self-cleaning performance. The higher WCA of 126.03° exhibited by the TSL with 5 wt% PTFE-µPs and SiO_2_-NPs inclusions was compared to that of other TSLs with 10 and 15 wt% PTFE-µPs and SiO_2_-NPs inclusions, exhibiting WCAs of 116.17° and 120.08°, respectively. This enhanced hydrophobicity indicated a stronger resistance to water and a greater ability to repel contaminants, as illustrated in Figure 10a–c. Conversely, when considering the electrospun EPS/PTFE-µPs/SiO_2_-NPs nanofibrous TSL with heat treatment, it was observed that the TSL with 15 wt% PTFE-µPs and SiO_2_-NPs inclusions demonstrated the most effective self-cleaning performance among the other tested compositions, as depicted in Figure 10d–f. These findings substantiated the self-cleaning characteristics of TSL with 15 wt% PTFE-µPs and SiO_2_-NPs inclusions subjected to the heat-treatment procedure, as illustrated in Figure 11, which indicated the presence of a hierarchical micro/nanostructure on the heat-treated electrospun EPS/PTFE-µPs/SiO_2_-NPs nanofibrous TSL surface. Due to the presence of these hierarchical structures, the Fe_3_O_4_ contaminants have contact primarily with the elevated regions of the micro/nano-scale surface while avoiding air pockets within the grooves. This unique arrangement reduces the Van der Waals force between the particles and the TSL surface. The self-cleaning capability of the TSL could be attributed to the reduced adhesion between the TSL surface and the Fe_3_O_4_ contaminants, as well as the significant capillary force generated by water droplets, facilitating the easy removal of Fe_3_O_4_ contaminants and allowing them to slide off the TSL surface and become attached to the edge of the water droplets during the rolling-off process [56,57]. Overall, it is evident that the self-cleaning efficacy of the electrospun EPS/PTFE-µPs/SiO_2_-NPs nanofibrous TSL is influenced by both heat treatment and the concentration of micro/nanoinclusions.

### 3.6. Antibacterial Study

**Figure 12:**
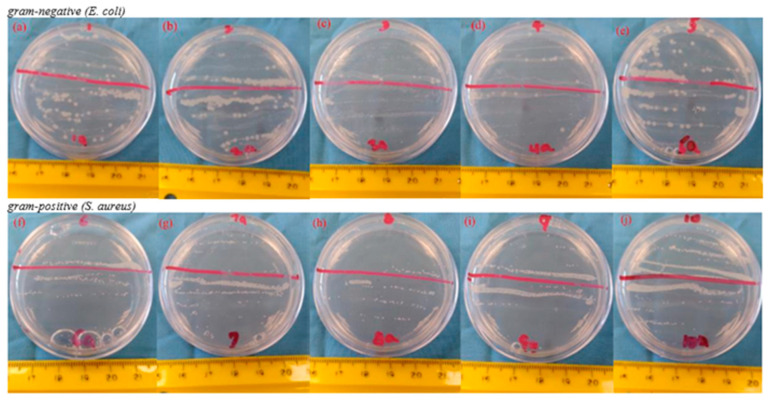
Images depicting bacteria survival following a 10 h incubation period of the electrospun MFL/HAp/Ag-NPs nanofibrous BHL with: (**a**,**f**) MFL with HAp and 0.1 wt% Ag-NPs; (**b**,**g**) MFL with HAp and 0.4 wt% Ag-NPs; (**c**,**h**) MFL with HAp and 0.8 wt% Ag-NPs; (**d**,**i**) MFL with HAp and 1.5 wt% Ag-NPs; and (**e**,**j**) MFL and HAp only.

**Figure 13:**
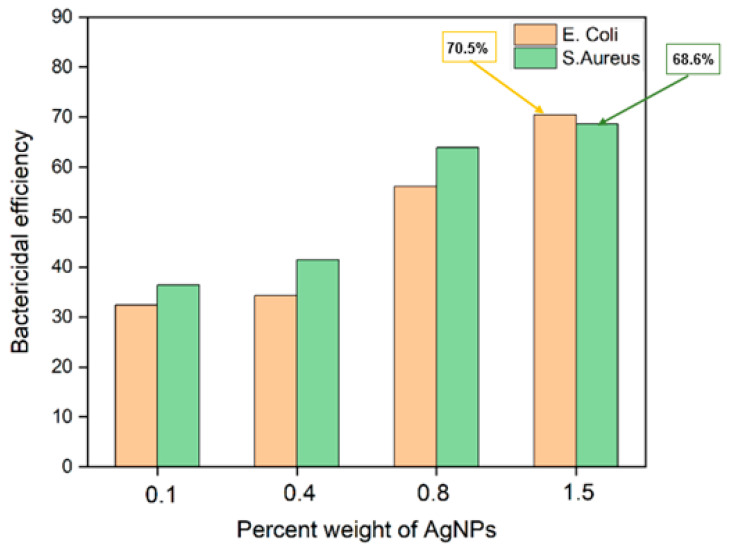
Efficiency of bacterial inhibition of electrospun MFL/HAp/Ag-NPs nanofibrous BHLs with HAp and different weight percentages of Ag-NPs against *E. coli* and *S. aureus*.

The antibacterial experiments were assessed to investigate the bactericidal properties of the electrospun MFL/HAp/Ag-NPs nanofibrous BHL of the tri-layered scaffold. We employed the bacterial viability counting approach to evaluate the effectiveness of BHLs against Gram-negative (*E. coli*) and Gram-positive (*S. aureus*) bacteria to determine the extent of bacterial growth inhibition by comparing the bacterial counts for each BHL (Figure 12). The electrospun MFL/HAp/Ag-NPs nanofibrous BHLs without Ag-NPs (0.0 wt%) were carried out as a control group to compare the antibacterial performance of other electrospun MFL/HAp/Ag-NPs nanofibrous BHLs with various weight percentages of Ag-NPs (0.1, 0.4, 0.8, and 1.5 wt%). The antibacterial efficacy of the BHLs fabricated with Ag-NPs increased noticeably with the increases in weight percentages of Ag-NPs, as illustrated in Figure 13. Specifically, the bacterial growth inhibition efficiencies of BHLs for *S. aureus* at 0.1, 0.4, and 0.8 wt% Ag-NPs are 36.4%, 41.4%, and 63.9%, respectively. Similarly, for *E. coli* at 0.1, 0.4, and 0.8 wt%, the Ag-NPs are 32.4%, 34.3%, and 56.2%, respectively. Furthermore, it was found that the highest bacterial growth inhibition efficiency of electrospun MFL/HAp/Ag-NPs nanofibrous BHLs for both *S. aureus* and *E. coli* are observed at 1.5 wt% Ag-NPs. At 1.5 wt% Ag-NPs, the bacterial growth inhibition efficiencies are 68.6% and 70.5% for *S. aureus* and *E. coli*, respectively (Figure 13).

Traditional chronic wound dressings often rely on small-molecule antibacterial agents, which offer the advantage of rapid sterilization. However, it is essential to acknowledge that these agents permeate the human body and exert adverse effects on human health [58]. The Ag-NPs have remarkable antibacterial properties and demonstrate excellent biocompatibility. HAp was also chosen as a critical component for this study because of its excellent biocompatibility, superior water absorption capabilities, and inherent hydrophilic properties [59,60,61]. Integrating these nanoparticles into the BHL of the tri-layered scaffold offers an effective resolution to hinder bacterial proliferation at the wound surface. The WCA and wettability results from this study show that the electrospun MFL/HAp/Ag-NPs nanofibrous BHLs exhibit excellent hydrophilicity, and the finding from the antibacterial study indicates that incorporating a small concentration of Ag-NPs (about 1.5 wt%) can significantly enhance the bacterial growth inhibition of electrospun MFL/HAp/Ag-NPs nanofibrous BHLs made from recycled MFL.

### 3.7. In Vitro Cytotoxicity Study

**Figure 14:**
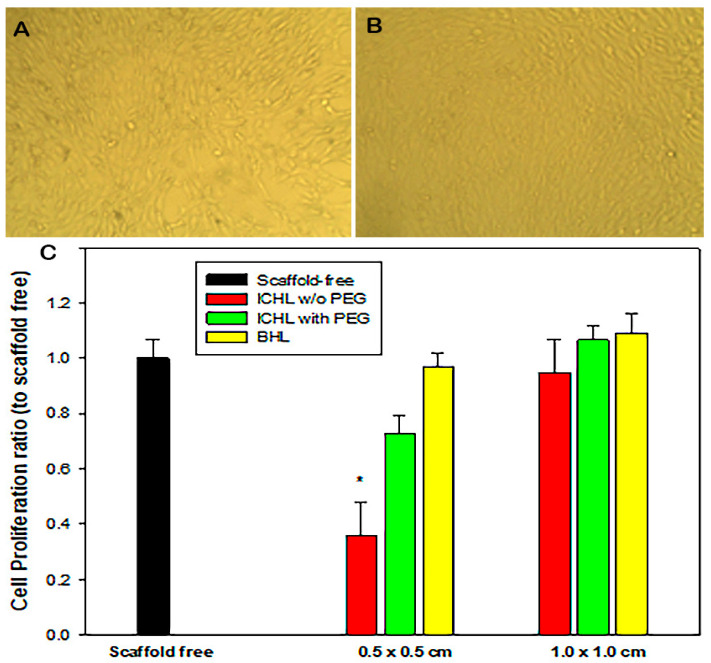
MTT cell viability after four days of co-culturing different sizes of the scaffold samples: (**A**) Representing cell culture without scaffold, and (**B**) a cell culture image with 1.0 cm^2^ nanofiber sample. (**C**) Quantification of cell proliferation ratios of the BHL and ICHL samples in comparison to the cell cultures without scaffold sample (* *p* < 0.05).

The basic property requirement of the tri-layered scaffold is high cytocompatibility. Therefore, the biocompatibility of the tri-layered scaffold was assessed by the colorimetric technique of reduction in MTT to formazan crystals by viable high metabolic cells. Figure 14 summarized the biocompatibility natures of the electrospun MFL/HAp/Ag-NPs nanofibrous BHLs and ICHL samples with or without PEG after cocultured with primary fibroblastic cells for four days. It is clearly shown that there were no evident cytotoxicity effects on the various sized BHL samples. Indeed, the porous structure of the MFL/HAp/Ag-NPs BHL of the tri-layered scaffold closely resembled the ECM, thereby providing a conducive environment for the growth and proliferation of fibroblastic cells.

Figure 14A,B exhibit some representative cell culture patterns of scaffold-free control and the one with a 1.0 cm^2^ BHL scaffold sample. No evident cell proliferation difference was noticed. MTT assay confirmed the biocompatibility of the BHL and ICHL samples and suggested that the middle layer samples cross-linked with PEG markedly increased cytocompatibility when compared to the ones without PEG (Figure 14C). It was unexpected that the size difference of the ICHL influenced the cell metabolic activities when there was no PEG in the layer. The increase in the sample areas improved cell survival, and the cross-link with PEG totally corrected the cytotoxicity. We postulate that the tri-layered scaffolds with the ICHL sample cross-linked with PEG are more conducive to cell growth and have better cell viability. Also, larger size of the tri-layered scaffold provides more bioactive components, such as BSA and aloe vera, as well as HAp in the electrospun MFL/HAp/Ag-NPs nanofibrous BHLs. These components promote cell growth and proliferation [62,63]. As highlighted by Lotfi et al., every cell possesses distinct requirements in terms of physical architecture and chemical composition, requiring the creation of a unique environment for optimal cellular function [64]. In this regard, the architecture of tri-layered scaffolds exhibited a notable advancement due to the ability to replicate the ECM. Therefore, providing a well-established network that facilitates Fibroblast adhesion, migration, and proliferation becomes an essential aspect of this promising tri-layered scaffold. In summary, developed tri-layered scaffolds with the ICHL sample cross-linked with PEG were found to have high cytocompatibility and be noncytotoxic to fibroblastic cells and most suitable for chronic wound healing [65,66].

## 4. Conclusions

In conclusion, this study fabricated a tri-layered scaffold composed of a top superhydrophobic layer, an intermediate chitosan hydrogel layer, and a bottom hydrophilic layer, demonstrating promising characteristics for wound healing applications. This tri-layered scaffold mimics some characteristics of full-thickness skin that offers a dense superficial top layer, bioactive intermediate layer, and porous bottom layer, all of which work synergistically to facilitate the chronic wound-healing process. In addition, the study also revealed that as the size of the tri-layered scaffold increased, so did the cell proliferation performance of the different scaffolds. Notably, the tri-layered scaffold with ICHL cross-linked with PEG enhanced cell proliferation. Further studies are on the way to quantitatively examine the antimicrobial effectiveness, wound healing progress, and long-term safety issues. Indeed, in vitro assessments and animal trials are warranted to validate its full potential for practical clinical application. The breakthroughs from this study could contribute to developing cost-effective and highly efficient materials tailored explicitly for chronic wound healing.

## Figures and Tables

**Table 1:** Scale-size effects among different layers comprising tri-layered scaffold.

Scale-Size	Electrospun EPS/PTFE-µPs/SiO_2_-NPs Nanofibrous TSL (cm^2^)	ICHL Sample Cross-Linked with and without PEG (g)	Electrospun MFL/HAp/Ag-NPs Nanofibrous BHL (cm^2^)
Whole	1.0	0.4	1.0
Half	0.5	0.2	0.5
Quarter	0.25	0.1	0.25

**Table 2:** FTIR peaks associated with spectra of tri-layered scaffold, as shown in Figure 6.

Peak Number	Wavenumber (cm^−1^)	Vibrational Mode
1	698.04	out-of-phase deformation of monosubstituted aromatic rings
2	757.49	strong C–Cl stretching
3	1097.08	strong C–O stretching of secondary alcohol
4	1652.92	medium C=C stretching of vinylidene
5	2190.10	weak C≡C stretching of alkyne
6	2923.60	medium C–H stretching of alkane
7	532.80	strong C–Br stretching of halo compound
8	752.40	strong C–H bending of monosubstituted compound
9	1083.92	strong C–O stretching of primary alcohol
10	1540.83	strong N–O stretching of nitro compound
11	1646.85	medium C=C stretching of conjugated alkene
12	2034.86	strong N=C=S stretching of isothiocyanate
13	2900.30	medium C–H stretching of alkane
14	3298.06	strong and broad O–H stretching of carboxylic acid
15	1091.90–1091.65	strong C–O stretching
16	1414.75–1415.88	strong S=O stretching of sulfate
17	1551.54–1557.51	strong N–O stretching of nitro compound
18	1636.53–1644.97	medium C=C stretching of cyclic alkene
19	3320.39–3351.24	strong O–H stretching

## Data Availability

All data supporting this study’s findings are available from the corresponding author upon reasonable request.
